# Determinants of late initiation for antenatal care follow up: the case of northern Ethiopian pregnant women

**DOI:** 10.1186/s13104-018-3938-9

**Published:** 2018-11-27

**Authors:** Fitsum Wolde, Zerfu Mulaw, Tibeb Zena, Belete Biadgo, Miteku Andualem Limenih

**Affiliations:** 1 Department of Midwifery, College of Health Science, Arbaminch University, Arbaminch, Ethiopia; 2 Department of Midwifery, College of Medicine and Health Science, University of Gondar, P.O. Box 196, Gondar, Ethiopia; 3 Department of Clinical Chemistry, College of Medicine and Health Science, University of Gondar, P.O. Box 196, Gondar, Ethiopia

**Keywords:** Antenatal care, Ethiopia, Late initiation of antenatal care

## Abstract

**Objective:**

Early antenatal care follow-up is the main strategy of preventing pregnancy related adverse outcomes; in which World Health Organization recommends first antenatal care visit should be offered within the first trimester. However, Low utilization and late booking is the predominant problem in most developing countries including Ethiopia. This study aimed to determine the prevalence of late initiation for antenatal care follow-up and associated factors among pregnant women. Institutional based cross-sectional study was conducted among 423 pregnant mothers using systematic sampling technique. Multivariable logistic regression analysis was performed at the level of significance of p-value ≤ 0.05.

**Results:**

The findings showed 59.4% of pregnant women started their first visit after first trimester. Having age ≥ 25 years (AOR = 1.62, CI 1.1, 2.49), recognition of pregnancy by missed period (AOR = 2.54 CI 1.63, 3.96), pregnant mother who were not advised to start antenatal-care (AOR = 3.36, CI 1.74, 6.5) and primary educational level (AOR = 2.22, CI 1.16, 4.25) were found to be significantly associated with late initiation for antenatal care. The prevalence of late antenatal care follow-up is high. Multidisciplinary approaches to keep empowering women through education are recommended for early initiation of antenatal care.

## Introduction

Sub-Saharan Africa including Ethiopia had the highest maternal mortality ratio of 510 and 420 per 100,000 live births respectively [1]. To alleviate this problem, antenatal care a key entry point. It’s a care given to pregnant mother from the time of conception until the beginning of labor to avoid and minimize possible risks of morbidity and mortality [2, 3]. Hence, World Health Organization recommends that a pregnant woman should have at least four antenatal care visits, the first of which should be on the first trimester [2].

Worldwide there is a big discrepancy in the prevalence of late ANC follow up among pregnant mothers, ranging from 27.5 to 88% in developed and developing countries respectively [4, 5]. Low prenatal coverage, few visits, and delayed initiation of antenatal follow up are the predominant problems throughout SSA including Ethiopia resulting in failure of accomplishment of the WHO recommendation [2].

The minimum ANC visits recommended by WHO was possible only for less than about a third of the pregnant women in some SSA countries like Niger (15%), Ethiopia (19%), Chad (23%), Burundi (33%), Mali and Rwanda (35%) [6].

Despite ANC is provided free of charge and there is increased accessibility; low utilization and late booking is still a major problem [7, 8]. According to Ethiopian Demographic Health Survey 2014, only 17.5% mothers start ANC early as per the recommendation [9].

Prevalence of late booking vary from country to country, not only because of its real difference in occurrence but also due to differences in definitions of late booking [3, 6]. According to researches, maternal age, maternal education, husband’s education, income, women’s employment, type of pregnancy, counseling on early initiation of ANC, Previous ANC service utilization, parity and means of pregnancy recognition are contributing factors [5, 10–24].

However, the determinants of late initiation for ANC are not the same across different cultures, socio economic status and access to institution within a society. Thus assessing the factors of late initiation of ANC follow up in different set up is important to improve maternal health services. Therefore, this study aimed at determining the prevalence of late ANC initiation and factors associated with it among pregnant women attending antenatal clinic in governmental health institutions at Debremarkos town, Ethiopia.

### Theoretical frame work

Maternal age, marital status, occupation, income, educational status and paternal education were the predisposing factors for late initiation of antenatal care follow-up whereas; previous antenatal care utilization, family size, means of pregnancy recognition and previous pregnancy complication were enabling factors and parity, type of pregnancy and knowledge on antenatal care visit were the need factors.

## Main text

### Study area and period

The study was conducted at Debremarkos governmental health institutions from March 1 to June 1, 2016. The total population of the town was 101,582 [28]. There are four governmental health institutions (three health centers and one referral hospital). To provide the service there were 1BSc, 14 diploma Midwives and 8 diploma Nurses.

### Study design

Institutional based cross sectional study design was employed.

### Study population

All pregnant women attending ANC in Debremarkos governmental health institutions during the study period were included.

### Sample size determination

A total of 423 samples were calculated using single population proportion formula by considering, 95% confidence level, 5% margin of error and the prevalence of late initiation for ANC follow up 52.6% [20] and considering 10% of possible non response rate.

### Sampling procedure

To select participants, systematic random sampling techniques were employed. The total sample size was proportionally allocated for three health centers and one referral hospital based on their ANC loads. By considering; N (total pregnant woman who came for first ANC visit in previous 3 months in 4 health institutions = 850), n (calculated sample size = 423) and k-interval (K = N/n, = 850/423 = 2), the first client was selected by lottery method among the first two ANC service users.

$${\text{n}}_{\text{h}} = {\text{N}}_{\text{h}} *{\text{n}}/{\text{N}}$$

[n_h_ = sample size allocated to each health institution, N_h_ = number of ANC clients in a health institution in previous 3 months (Debremarkos referral hospital = 280, Wuseta health center = 220, Hidase Health center = 210 and Debremarkos Health center = 140), N = cumulated number of ANC clients in all health institutions in previous 3 months (850), n = total sample size (423)] resulting in final sample size (Debremarkos referral hospital = 139, Hidase health center = 105, Wuseta Health center = 109 and Debremarkos health center = 70).

### Construction of variables

Late initiation for ANC visit was the outcome variable where as the independent variables were constructed in demographic and socio-economic variables (maternal age, marital status, education, occupation, income, family size, paternal educational status), obstetric related factors (parity, means of pregnancy recognition, type of pregnancy, previous history of complication), service related variables (previous utilization of ANC service) and client related variables (advice from others, Knowledge about ANC).

### Data collection instruments and techniques

Data was collected by face to face interview using structured and pre-tested questionnaire. The questionnaire was first prepared in English and translated to local language for interview. The data was collected by eight diploma Midwives.

### Data processing and analysis

Data were entered into EPI Info version 7.1.2.0 and exported to SPSS version 20 software for analysis. Variables having a p-value of ≤ 0.20 in bivariate analysis were entered into multiple logistic regressions. Finally variables with a p-value of ≤ 0.05 were considered as statistically significant. Moreover, multi-colinearity was checked using the variance inflation factor, and it shows no colinearity effect.

### Socio-demographic characteristics of the participants

A total of 423 mothers who came for ANC follow up in Debremarkos governmental health institutions were interviewed with a response rate of 98.35%. The median age was 25 years. Majority of them, 405 (97.4%) were Orthodox Christians. Amhara ethnic group holds 415 (99.8%) (Table 1).

**Table 1:** Socio-demographic and economic characteristic of pregnant mother attending ANC service in Debre Markos governmental health institution, Ethiopia, 2016 (n = 416)

Variables	Frequency (%)
Age	
< 25 (young)	180 (43.3%)
≥ 25	236 (56.7%)
Ethnicity	
Amhara	415 (99.8%)
Tigrie	1 (0.2%)
Marital status	
Married	396 (95.2%)
Cohabitation	11 (2.6%)
Divorced/widowed	9 (2.2%)
Religion	
Orthodox	405 (97.4%)
Muslim	6 (1.4%)
Protestant	5 (1.2%)
Educational status	
No formal education	103 (24.8%)
Primary [1–8]	82 (19.7%)
Secondary [9, 10] and certificate	125 (30%)
College diploma and above	106 (25.5%)
Employment status	
Employed	254 (61.1%)
Not employed	162 (38.9%)
Father education	
No formal education	62 (14.9)
Primary [1–8]	78 (18.8)
Secondary [9, 10] and certificate	119 (28.6)
College diploma and above	157 (37.7%)
Family monthly income	
< US$37	50 (12%)
$US37–US$148	284 (68.3%)
> US$148	82 (19.7%)
Family size	
≤ 3	286 (68.8)
> 3	130 (31.2%)

### Obstetric history related characteristics of the study subjects (n = 416)

Two hundred forty-nine (59.9%) of pregnant women were multigravida; out of this, 179 (71.9%) had no history of complication during their last pregnancy. 168 (40.4%), 167 (40.1%) and 81 (19.5%) were primiparous, nulliparous and multiparous respectively.

### Knowledge of participants about ANC service

Three hundred eighty-nine (93.5%), 16 (3.9%) and 11 (2.6%) participants rated the importance of ANC follow up as highly, medium and less important for both the mother and fetus respectively. Of the respondents, 187 (45%), 188 (45.1%) and 41 (9.9%) reported that the right time to start ANC follow up is in the first 3 months of pregnancy, after 3 months of pregnancy and they don’t know the right time of initiation respectively. Three hundred nine (74.3%) knows number of ANC visit should be ≥ 4.

### Past history of ANC service utilization

Among 249 (59.9%) pregnant women who had a history of previous pregnancy, 69.9% of them had ANC follow up for the previous last pregnancy. Among these, 48.8% of them had their previous ANC visit at 12 weeks.

### Pregnant mother’s commencement to ANC follow-up

In this study, 59.4% of participants started their ANC follow up lately. Among the late comers for ANC follow up, 43.3% of them perceived that it is the right time to start, while other mentioned reasons are; previous experience (26.7%), lack of time (14.2%), unplanned pregnancy (8.9%), because of sickness and unawareness of pregnancy (6.5%) and negligence (0.4%).

**Fig. 1:**
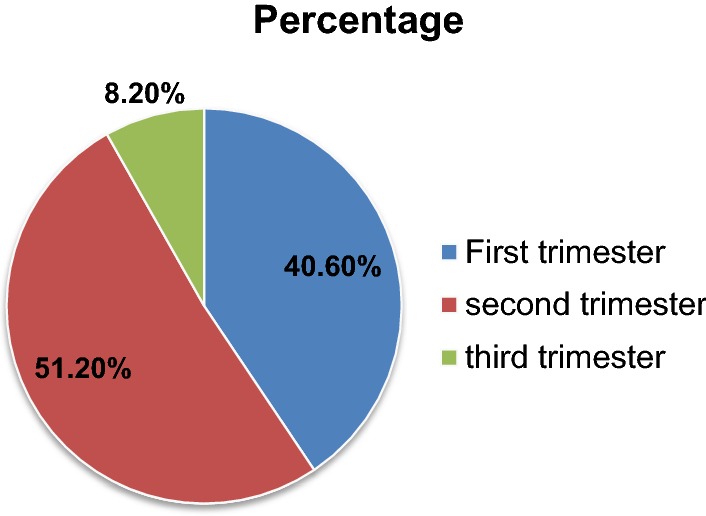
Time for first ANC follow up of study participants in governmental health institutions at Debremarkos, Ethiopia, 2016

The timing of first ANC booking ranged from week 4 to 40; the median gestational age during the first antenatal care booking was 15 weeks (Fig. 1).

### Associated factors for late ANC follow up initiation

Maternal age, means of pregnancy recognition, maternal educational status, advice to start visit were significant factors for late ANC follow up initiation. The odds of pregnant mother aged 25 years and above were 1.62 times more likely to have a late initiation for ANC follow up than their counter parts (AOR = 1.62, CI 1.049, 2.49). Those pregnant mothers who recognized their pregnancies by missed period were 2.54 times more likely to have late ANC initiation than those recognized their pregnancy by urine test (AOR = 2.54 CI 1.63, 3.96). Pregnant mothers who were not advised to start ANC were 3.36 times more likely to come late for ANC follow up than those who received advice (AOR = 3.36, CI 1.74, 6.5). Mothers who attended primary education were 2.22 times more likely to have late ANC follow-up initiation than those mothers had diploma and above (AOR = 2.22, CI 1.16, 4.25) (Table 2).

**Table 2:** Logistic regression showing factors on late ANC follow up initiation among mothers who came for ANC clinic in Debremarkos governmental health institution, Ethiopia, 2016 (n = 416)

Variable	Late initiation		OR (95% CI)	
	Yes	No	COR	AOR
Maternal age				
< 25 (young)	97 (53.9%)	83 (46%)	1	1
≥ 25	150 (63.6%)	86 (36.4%)	*1.49 (1.005, 2.22)*	*1.62 (1.049, 2.49)*
Maternal educational status				
No formal education	60 (58.3%)	43 (41.7%)	1.62 (0.94, 2.8)	^a^
Primary (1–8)	58 (70.7%)	24 (29.3%)	*2.8 (1.53, 5.2)*	*2.22 (1.16, 4.25)*
Secondary and certificate	80 (64%)	45 (36%)	2.1 (1.23, 3,5)	^a^
College diploma and above	49 (46.2%)	57 (53.8%)	1	1
Employment status				
Not employed	104 (64.2%)	58 (35.8%)	1.39 (0.93, 2.1)	^a^
Employed	143 (56.3%)	111 (43.7%)	1	1
Knowledge on ANC				
Inadequate knowledge	22 (73.3%)	8 (26.7%)	1	1
Adequate knowledge	225 (58.3%)	161 (41.7%)	1.97 (0.9, 4.5)	^a^
Family size				
> 3	70 (53.8%)	60 (46.2%)	0.72 (0.5, 1.1)	^a^
≤ 3	177 (61.9%)	109 (38.1%)	1	1
Income				
< 1000 (Q1)	30 (60%)	20 (40%)	1.65 (0.8, 3.4)	^a^
1000–4000	178 (62.7)	106 (37.3%)	1.85 (1.13, 3.04)	^a^
> 4000 (Q3)	39 (47.6%)	43 (52.4%)	1	1
Pregnancy recognition				
By missed period	164 (69.5%)	72 (30.5%)	*2.7 (1.8, 3.98)*	*2.54 (1.63, 3.96)*
By urine test	83 (46.1%)	97 (53.9%)	1	1
Advise to start ANC visit				
No	53 (79%)	14 (20.9%)	*3 (1.62, 5.66)*	*3.36 (1.74, 6.5)*
Yes	194 (55.6%)	155 (44.4%)	1	1
Type of pregnancy				
Not planned	40 (71.4%)	16 (28.6%)	1.85 (0.9, 3.4)	^a^
Planned	207 (57.5%)	153 (42.5%)	1	1
Paternal educational status				
No formal education	37 (59.7%)	25 (40.3%)	1.32 (0.73, 2.4)	^a^
Primary (1–8)	52 (66.7%)	26 (33.3%)	1.8 (1, 3.1)	^a^
Secondary and certificate	75 (63%)	44 (37%)	1.5 (0.9, 2.5)	^a^
College diploma and above	83 (52.9%)	74 (47.1%)	1	1

The italicized value indicated that a statistically significant association at 95% Confidence interval(CI) that did not include 1 in the interval

1 = reference category

^a^Not significant in stepwise backward logistic regression. Hosmer and Lemanshow test for multivariable log reg. = 0.85

### Discussion

This study revealed that 59.4% of pregnant women started their ANC follow up after 12 weeks of gestation, which is late ANC follow up initiation. This finding is in line with a study done in Addis Ababa 59.8% [11].

The finding of this study was encouraging as compared to the studies conducted in Uganda (83%), Nigeria (82%) and Thailand (73.8%) [8, 25, 26]. However, it is remarkably higher than the study done in Vietnam in which late ANC follow up were 27.5% [4]. This might be due to the difference in socioeconomic status and health service coverage. The difference in prevalence across studies might be socio demographic features of the study participants, media of information among health care givers, knowledge of mothers on importance of early ANC follow up, the time gap between the study periods and the difference in data collection methods [4, 8].

Pregnant mother aged ≥ 25 were 1.62 times more likely to book late than those pregnant women age less or equal to 25 years. This result is supported by the studies done in Nigeria, Kembata and Gondar [16–18]. This might be due to women in the older age group are more likely to have many children to care and many of the older pregnant women’s might have ingrained cultural biases against formal health care [16].

On the contrary, studies done in rural Adigrat, Uganda and Vietnam showed that teenagers were more likely to start ANC lately than adults [27–29]. This observed difference might be due to the difference in the study population, in which all studies included rural population. Its fact that rural setup can generally be expected to have less knowledge and experiences about ANC.

Pregnancies, recognized by missed periods were 2.54 times more likely to initiate ANC lately than those who recognized by a urine test. This finding is consistent with the study done in Gondar [18]. This might be due to the reason that mothers having irregular menstrual cycle are not curious about the missed period. This is supported by the study done in South African women that delayed recognition of pregnancy and late ANC initiation has also been reported [30].

Those pregnant mothers who were not counseled to start ANC follow-up were 3.36 times more likely to have late initiation of ANC follow up than those who received counseling. This result is in line with the study done in Adigrat and Nigeria, in which those pregnant mothers who were advised by health workers started their ANC follow up timely [10, 25].

It might be due to the fact that provision of appropriate information changes health seeking behavior of women and motivate them to visit a well-organized health institution timely [5].

Respondents who were in primary educational status were 2.22 times more likely to initiate ANC lately than those having collage diploma and above. This result is supported by similar other study in South Sudan [31]. The possible explanation might be, as the level of educational status increases, the pregnant women would more likely to give value for the benefit of timely initiation. Furthermore, pregnant women who had college diploma and above have information on benefits of timely booking for ANC [5].

### Conclusions and recommendation

Late initiation of ANC is a high problem in the study area. Maternal age ≥ 25, means of pregnancy recognition, lack of advice, and being at primary educational status were determinant factors for late ANC initiation. Keeping strengthening the expansion of education on ANC, promoting health education, creating awareness, empowering women and giving appropriate advice on pregnancy and timely ANC follow-up would improve late initiation for antenatal care.

## Limitations of the study

Gestational age was determined based on women’s report. This might have inaccuracies in the measurement of gestational age.

## Abbreviations

ANC: antenatal care
AOR: adjusted odds ratio
CI: confidence interval
COR: crude odds ratio
EDHS: Ethiopian Demographic and Health Survey
SPSS: Statistical Package for Social Science
SSA: Sub-Saharan Africa
WHO: World Health Organization
